# From “Monarch, Minister, Assistant, and Envoy” to “Microbial Dialogue”: A Review of Novel Mechanisms by Which Chinese Herb Pairs Improve Metabolic Diseases Through Gut Microbiota Metabolic Regulation

**DOI:** 10.1155/grp/8226799

**Published:** 2026-06-15

**Authors:** Zeyu Wang

**Affiliations:** ^1^ The First Clinical Medical College, Nanjing University of Chinese Medicine, Nanjing, Jiangsu Province, China, njucm.edu.cn

**Keywords:** Chinese herb pairs, gut microbiota, gut–organ axis, metabolic diseases, metabolic regulation, microbial metabolites

## Abstract

Metabolic diseases, including obesity, Type 2 diabetes, and nonalcoholic fatty liver disease, have become a severe global health burden, and their pathogenesis is closely associated with gut microbiota dysbiosis. As the core unit of traditional Chinese medicine (TCM) compatibility theory, Chinese herb pairs possess unique advantages in metabolic regulation due to their multicomponent and multitarget characteristics. Recent studies have confirmed that herb pairs can improve host metabolic homeostasis by reshaping gut microbial community structure, regulating microbial metabolites (e.g., short‐chain fatty acids, bile acids, and tryptophan metabolites), and repairing intestinal barrier function. This review systematically summarizes the latest research progress regarding the intervention of Chinese herb pairs in metabolic diseases by modulating the gut microbiota metabolic network, provides an in‐depth analysis of their action mechanisms based on the “microbiota–gut–organ axis,” and discusses the current research challenges and future translational directions.

## 1. Introduction

Metabolic diseases, including obesity, Type 2 diabetes mellitus (T2DM), nonalcoholic fatty liver disease (NAFLD), and cardiovascular disease (CVD), are major global health challenges, with insulin resistance and disordered glucose and lipid metabolism as core pathological features [1–3]. Driven by genetic predisposition, environmental factors, and lifestyle changes, the global prevalence of these diseases is rising sharply [3, 4]. Insulin resistance is the central pathological hub, triggering a series of metabolic disorders such as hyperglycemia, hyperinsulinemia, and atherogenic dyslipidemia (elevated triglycerides, decreased high‐density lipoprotein cholesterol, and increased small dense low‐density lipoprotein) [1], [2], [5]. This metabolic disorder is closely linked to chronic low‐grade inflammation and endothelial dysfunction, forming a vicious cycle that promotes disease progression [5, 6]. Therefore, insulin resistance and dyslipidemia are closely related to the occurrence of metabolic syndrome, T2DM, NAFLD, and polycystic ovary syndrome (PCOS) and significantly increase the risk of cardiovascular morbidity and mortality [1], [3], [5], [7]. Traditional treatment strategies mostly focus on single metabolic pathways or endpoints (such as lowering blood glucose or lipids), but the complex and interconnected characteristics of metabolic diseases give this reductionist approach inherent limitations and prevent it from addressing the underlying systemic metabolic disorders [8].

In recent years, the human gut microbiota, known as the “second genome,” has been recognized as a critical environmental factor and dynamic metabolic organ, which profoundly affects host physiology and disease susceptibility [7, 9]. A large number of preclinical and clinical studies have confirmed that gut microbiota dysbiosis is closely associated with the pathogenesis of obesity, T2DM, NAFLD, CVD, and other metabolic diseases [10], [11], [12]. Dysbiosis can damage intestinal barrier integrity, increase intestinal permeability, and cause bacterial components such as lipopolysaccharide (LPS) to enter the systemic circulation, resulting in metabolic endotoxemia, which triggers chronic inflammation and insulin resistance [10, 13]. In addition, the gut microbiota directly regulates host metabolism through its metabolites. Key microbial metabolites such as short‐chain fatty acids (SCFAs) produced by dietary fiber fermentation, secondary bile acids, trimethylamine N‐oxide (TMAO), and tryptophan metabolites act as important signaling molecules [11, 12, 14], regulating energy homeostasis, glucose and lipid metabolism, immune response, and neuroendocrine signaling, making the gut microbiota a core regulator at the interface of diet, metabolism, and immunity [14, 15]. For example, SCFAs (such as butyrate) have anti‐inflammatory and insulin‐sensitizing effects, while TMAO is related to atherosclerosis [16, 17]. The complex host–microbiota–metabolite interaction network forms the “gut–organ axis” (including gut–liver, gut–brain, and gut–heart axes), through which the intestinal ecosystem communicates with and regulates distant organs [17–19]. Therefore, targeting the gut microbiota to restore ecological balance and normalize metabolic function has become a new holistic therapeutic strategy for metabolic diseases.

With a history spanning thousands of years, traditional Chinese medicine (TCM) has a unique theoretical and practical system for disease treatment, emphasizing holistic regulation and syndrome differentiation. The core of TCM theory is the compatibility of multiherb formulas, which follow the “Jun–Chen–Zuo–Shi” (Monarch–Minister–Assistant–Envoy) principle to achieve synergistic effects and reduce toxicity [20, 21]. Herb pairs, as the simplest and most basic unit of TCM formulas, embody the essence of TCM compatibility, aiming to enhance efficacy, reduce side effects, and target multiple pathological links simultaneously [22, 23]. Modern pharmacological studies have verified the scientific connotation of TCM compatibility: classic herb pairs such as *Salvia miltiorrhiza* (Danshen)–*Carthamus tinctorius* (Honghua) and *Rheum palmatum* (Dahuang)–*Coptis chinensis* (Huanglian) have better therapeutic effects than single herbs [24, 25]. Notably, TCM and modern microbiome science have found an important intersection: most bioactive components in Chinese herbs (polyphenols, flavonoids, alkaloids, polysaccharides, etc.) are poorly absorbed in the upper gastrointestinal tract and reach the colon in large quantities, becoming substrates for gut microbiota metabolism [26, 27]. The gut microbiota can biotransform these herbal components into more bioavailable active metabolites (such as deglycosylation of flavonoid glycosides) [26–28]; conversely, herbal components can selectively regulate gut microbiota composition (promote beneficial bacteria, such as *Akkermansia*, *Lactobacillus*, and *Bifidobacterium*, and inhibit pathogenic bacteria) and enhance intestinal barrier function [29], [30], [31], forming a dynamic “herb–microbiota–host” interaction network, through which TCM interventions improve metabolic disorders by remodeling the gut microbiota ecosystem and its metabolic output [32, 33].

This review systematically summarizes the latest research on the mechanisms by which TCM herb pairs improve metabolic diseases by interacting with the gut microbiota and analyzes these mechanisms from multiple dimensions: (1) the regulatory effect of herb pairs on key microbial metabolites (SCFAs, bile acids, TMAO, and tryptophan metabolites) and their role in regulating host glucose and lipid metabolism, inflammation, and insulin sensitivity [16], [18], [34]; (2) the effect of herb pairs on strengthening intestinal mucosal barrier function, reducing endotoxemia, and regulating immune response, thereby alleviating chronic inflammation in metabolic disorders [10, 13]; (3) the multiorgan crosstalk mediated by the gut microbiota (gut–liver, gut–brain, and gut–heart axes) and the systemic metabolic benefits induced by herb pairs [21], [22], [35]; and (4) the challenges and translational prospects of targeting the “herb–microbiota–metabolism” axis to develop TCM strategies for preventing and treating metabolic syndrome. By integrating the ancient “Jun–Chen–Zuo–Shi” theory with the modern “microbial dialogue” concept, this review aims to provide a new theoretical basis and innovative therapeutic strategies for addressing the global epidemic of metabolic diseases.

## 2. Interaction Basis Between TCM Herb Pairs and Gut Microbiota

### 2.1. Microbial Metabolism and Biotransformation of TCM Components

The therapeutic effect of most TCM components depends on the biotransformation of gut microbiota because polysaccharides, flavonoids, alkaloids, saponins, and other components have low bioavailability and are difficult for the host to absorb in their original form [26]. This presystemic metabolism is mediated by various microbial enzymes (glycosidases, esterases, reductases, etc.) that catalyze hydrolysis, reduction, dehydroxylation, deglycosylation, and other reactions [29], converting complex parent molecules into smaller, more active, and more readily absorbed metabolites (such as aglycones), which are the real pharmacodynamic material basis of herbs [26]. For example, berberine, a key component of *C. chinensis* (Huanglian) and its herb pair *C. chinensis*–*Evodia urticaria*, is biotransformed into dihydroberberine by gut microbiota, which has enhanced hypoglycemic and lipid‐lowering activities [36]. Similarly, polysaccharides from *Astragalus membranaceus* (Huangqi) and *Codonopsis pilosula* (Dangshen) are not directly absorbed but fermented by specific gut bacteria, decomposed into oligosaccharides and monosaccharides, and utilized by saccharolytic bacteria to produce beneficial metabolites such as butyrate [30]. This biotransformation is not limited to single components: complex TCM formulas such as Xian–Ling‐Gu–Bao (XLGB) can be metabolized by rat intestinal flora to convert asperosaponin VI, epimedin C, and other glycosides into aglycones [31]. This microbial processing is a key chemical prerequisite, connecting the oral administration of complex herbal materials and their systemic biological effects, making the gut microbiota an indispensable mediator of TCM pharmacodynamic effects [29].

### 2.2. Remodeling of Gut Microbiota Community Structure by Herb Pairs

TCM herb pairs can significantly affect the composition and structure of gut microbiota, with the dual effects of selectively inhibiting pathogenic bacteria and promoting the proliferation of beneficial bacteria, thereby correcting the dysbiosis related to metabolic diseases [29]. This remodeling relies on the antibacterial and prebiotic properties of the complex phytochemical mixture in herb pairs, which can significantly improve microbial alpha diversity and restore the balance of key bacterial phyla (such as the Firmicutes/Bacteroidetes ratio) disturbed in obesity and T2DM [37]. For example, the herb pair *Pueraria lobata*–*Scutellaria baicalensis* (Gegen–Qinlian) improves metabolic disorders by regulating the gut microbiota community [37]. The structural remodeling induced by herb pairs is dose‐dependent and compatibility‐specific, and the combined effect of two herbs is better than the sum of the effects of the single herbs [38]. Comparative studies in colitis models show that both tonic and heat‐clearing herbs can improve dysbiosis, but their effects on microbial flora and host metabolism are different, and herb pairs have a more comprehensive regulatory effect [38], embodying the scientific connotation of TCM compatibility. In addition, herb pairs can enrich specific beneficial genera: the Shenling Baizhu powder (SLBZS) can increase *Akkermansia*, *Lactobacillus*, and *Muribaculum* and inhibit conditional pathogenic bacteria [34]; Gushudan (a kidney‐yang deficiency formula) can promote butyrate‐producing bacteria (Lachnospiraceae NK4A136 group) and lactate‐producing *Lactobacillus* [35]. These beneficial communities produce SCFAs and other metabolites to improve intestinal barrier function and regulate metabolism [39], and the remodeled microbiota can further biotransform herbal components, forming a positive feedback loop to amplify the therapeutic effect [29]. This targeted regulation of gut ecosystem structure and function is the core mechanism of systemic metabolic improvement by TCM herb pairs.

## 3. Regulating Microbial Metabolites: Core Mechanism

### 3.1. Regulation of SCFA Metabolism

TCM herb pairs rich in dietary fiber can act as prebiotics to promote the proliferation of SCFA‐producing bacteria (*Faecalibacterium*, *Roseburia*, *Lachnospira*, and *Ruminococcus*), increasing the production of acetate, propionate, and butyrate [40, 41]. SCFAs are important signaling molecules and energy substrates: butyrate is the main energy source of colonocytes, upregulating tight junction proteins (ZO‐1 and Occludin) to strengthen the intestinal barrier [42]; propionate and acetate enter the systemic circulation, regulating hepatic gluconeogenesis, lipid metabolism, and insulin sensitivity in the skeletal muscle and liver [41–43]. For example, the herb pair *Pinellia ternata*–*C. chinensis* (Banxia–Huanglian) for T2DM exerts hypoglycemic effects by increasing fecal butyrate and activating its receptors GPR43/GPR41 [44, 45], further inhibiting histone deacetylases (HDACs) and regulating the AMPK pathway, improving glucose homeostasis and reducing inflammation [46, 47]. In NAFLD, *Gynostemma pentaphyllum* saponins and *Astragalus* flavonoids (calycosin‐7‐O‐*β*‐D‐glucoside) alleviate hepatic steatosis and inflammation by enriching SCFA‐producing bacteria and activating PPAR*γ* and AMPK [48, 49]. SCFAs also regulate metabolism and immunity through the gut–heart and gut–brain axes [50, 51], becoming a core bridge connecting dietary fiber, microbial fermentation, and host physiological response [52, 53].

### 3.2. Reprogramming of Bile Acid Metabolism

TCM herb pairs can reprogram bile acid metabolism by regulating gut bacteria with bile salt hydrolase (BSH) activity (*Lactobacillus* and *Bifidobacterium*) [54]. These bacteria deconjugate primary bile acids (cholic acid and chenodeoxycholic acid), thereby changing the composition and ratio of the intestinal bile acid pool and increasing secondary bile acids (deoxycholic acid [DCA] and lithocholic acid [LCA]) or taurine‐conjugated bile acids [55]. The altered bile acid profile activates key receptors (farnesoid X receptor [FXR] and G protein–coupled bile acid receptor [TGR5]): FXR activation in the ileum and liver induces fibroblast growth factor 19 (FGF19) in humans (and FGF15 in mice), inhibits hepatic bile acid synthesis (CYP7A1), and regulates glucose and lipid metabolism, energy expenditure, and glucagon‐like peptide‐1 (GLP‐1) secretion [56, 57]; TGR5 activation in intestinal L‐cells stimulates GLP‐1 secretion, enhancing insulin secretion and improving glucose tolerance [45]. For example, the herb pair *Artemisia capillaris*–*Gardenia jasminoides* (Yinchen–Zhizi) for metabolic dysfunction–associated steatotic liver disease (MASLD) alleviates hepatic steatosis and inflammation by regulating gut microbiota, altering bile acid metabolism, inhibiting the intestinal FXR–FGF15 pathway, reducing bile acid synthesis, and promoting cholesterol excretion [58]. The Quyu Huazhuo decoction and Yuzhuo Zhixiao pill improve nonalcoholic steatohepatitis (NASH) by remodeling gut microbiota, increasing protective bile acids (ursodeoxycholic acid [UDCA]) and decreasing toxic DCA, activating beneficial FXR signaling [55–58]. *Lithospermum erythrorhizon* polysaccharide (LEP) exerts antiobesity effects by inhibiting BSH‐producing bacteria, increasing taurine‐conjugated bile acids, and activating adipose tissue thermogenesis [59]. The “microbiota–bile acid–FXR/TGR5 axis” is a key pathway for TCM herb pairs to protect the liver and regulate metabolism [60, 61].

### 3.3. Intervention in the Tryptophan Metabolic Pathway

TCM herb pairs can regulate the microbial metabolism of dietary tryptophan, affecting its three key metabolic pathways: indole derivatives (indole‐3‐propionic acid [IPA]), kynurenine pathway, and serotonin (5‐hydroxytryptamine [5‐HT]) precursor synthesis [62]. Indole derivatives are natural ligands of the aryl hydrocarbon receptor (AhR), and AhR activation enhances intestinal barrier function, reduces inflammation, and maintains immune homeostasis [63]. The kynurenine pathway is related to neuropsychiatric comorbidities (depression) of metabolic diseases, and the imbalance between kynurenine and serotonin pathways leads to neuroinflammation and depressive behavior [62]. TCM herb pairs with tranquilizing effects (such as *Polygala tenuifolia* [Yuanzhi]–*Poria cocos* [Fuling]) improve diabetic cognitive dysfunction by regulating the tryptophan–microbiota–brain axis. For example, Sini San (SNS) improves depression‐like behaviors in chronic stress models by regulating gut microbiota (increasing Firmicutes and Lactobacillaceae), promoting tryptophan, serotonin, and kynurenine production in the hippocampus and colon, and increasing SCFAs (butyrate and propionate) [62]. Studies on poststroke depression (PSD) show that decreased SCFAs (butyrate and acetate) are related to lipid metabolism disorders in the prefrontal cortex, suggesting the Firmicutes–SCFA–lipid metabolism axis as a potential pathway of the microbiota–gut–brain axis [64]. In addition, *Bletilla striata* oligosaccharides improve metabolic syndrome by reversing gut dysbiosis and restoring tryptophan metabolite homeostasis [65]; *Atractylodes macrocephala* polysaccharides alleviate ulcerative colitis by regulating tryptophan metabolism [66]. By regulating tryptophan microbial metabolism, TCM herb pairs simultaneously improve intestinal integrity, systemic inflammation, and neuropsychiatric symptoms of metabolic diseases, reflecting the interaction of the gut–brain axis [62–67].

## 4. Repairing Intestinal Barrier Function and Alleviating Metabolic Endotoxemia

### 4.1. Enhancing Physical Barrier Function

Metabolic diseases are often accompanied by increased intestinal permeability (“leaky gut”), damaging the physical barrier composed of intestinal epithelial cells and tight junction proteins, leading to the translocation of microbial products and metabolic endotoxemia [68], [69], [70], [71]. TCM herb pairs (such as Astragali Radix–Glycyrrhizae Radix and Codonopsis Radix–Atractylodis Macrocephalae Rhizoma) repair the intestinal barrier by regulating gut microbiota to increase SCFA production [72–76]. SCFAs are energy substrates for colonocytes, activate GPR43, GPR109A, AMPK, and mTOR pathways, promoting intestinal epithelial cell proliferation and differentiation [75–77], and upregulate tight junction proteins (ZO‐1, Occludin, and claudins) to enhance the paracellular barrier [68, 78, 79]. For example, the Danggui Buxue decoction (Astragali Radix–*Angelica sinensis*) and Paeoniae Radix Alba increase the expression of ZO‐1, Occludin, and Claudin‐1, improving barrier function [68, 69, 78, 79]. In addition, herb pairs enhance the chemical barrier by promoting goblet cells to secrete Mucin‐2 (MUC‐2), thickening the mucus layer and inhibiting pathogenic bacteria adhesion [68], [80, 81]. This multitarget regulation (increasing SCFAs, activating repair pathways, strengthening tight junctions, and enhancing mucus secretion) effectively restores intestinal barrier integrity, laying the foundation for improving systemic metabolic disorders (obesity, T2DM, and hyperlipidemia) [69], [82], [83].

### 4.2. Inhibiting Inflammation and Reducing Endotoxin Translocation

Gut dysbiosis in metabolic diseases leads to the overgrowth of Gram‐negative bacteria, increasing LPS production [73]. LPS translocates into the systemic circulation through the damaged intestinal barrier, causing metabolic endotoxemia, activating the TLR4/NF‐*κ*B pathway, and promoting the secretion of proinflammatory cytokines (TNF‐*α*, IL‐6, and IL‐1*β*), inducing insulin resistance and metabolic dysfunction [70, 71], [84–86]. TCM herb pairs (such as Coptidis Rhizoma–Rhei Radix et Rhizoma and Salviae Miltiorrhizae Radix–Carthami Flos) exert dual anti‐inflammatory effects: direct inhibition of the TLR4/NF‐*κ*B pathway by alkaloids (berberine) and phenolic acids [87], [84], [88] and indirect regulation of gut microbiota, promoting beneficial bacteria (*Lactobacillus*, *Bifidobacterium*, and *Akkermansia*) to produce SCFAs, inhibiting LPS‐producing pathogenic bacteria (Proteobacteria), and reducing endotoxin production at the source [68], [89, 90], [29], [91]. SCFAs further inhibit NF‐*κ*B activation and promote regulatory T cell (Treg) differentiation by inhibiting HDACs and activating GPRs [72–76]. Through the synergistic effect of direct anti‐inflammation and indirect microbiota regulation, herb pairs alleviate metabolic endotoxemia, improve insulin sensitivity, and inhibit the chronic inflammation underlying metabolic disease progression [83], [92, 93].

## 5. Regulating the “Microbiota–Gut–Organ Axis” Crosstalk

### 5.1. Core Role of the Gut–Liver Axis

The gut–liver axis is a bidirectional communication pathway between the liver and the intestine, which is severely disturbed in MASLD, leading to intestinal barrier dysfunction, microbial metabolite disorder, and gut–derived toxin translocation [70]. LPS and other toxins enter the portal circulation and activate hepatic Kupffer cells via TLR4, inducing chronic inflammation and hepatic steatosis [71]. Gut dysbiosis also disrupts nitrogen metabolism, increasing ammonia production and hepatic ammonia accumulation, aggravating liver injury and fibrosis [87]. Bile acid metabolism disorder is a hallmark of MASLD: dysbiosis affects BSH activity, altering bile acid pool composition and FXR signaling, regulating lipid and glucose metabolism [71]. The liver secretes bile acids to regulate gut microbiota, forming a feedback loop. TCM interventions targeting the gut–liver axis have significant effects: the JiGuCao capsule repairs the intestinal barrier (upregulating Claudin‐1 and Occludin), reduces serum LPS, increases *Lactobacillus*, and regulates hepatic lipid metabolism (PPAR*γ*–CD36) [82]; the herb pair *Alisma orientale*–*A. macrocephala* (Zexie–Baizhu) restores gut microbiota, enhances intestinal barrier function, regulates bile acid metabolism via the FXR pathway, and inhibits NLRP3 inflammasome and M1 macrophage polarization [94]; the Zhuyu pill regulates bile acid metabolism through the gut–liver axis, inhibits ileal FXR–FGF15 signaling, upregulates hepatic CYP7A1, and promotes cholesterol excretion [95]. *Akkermansia muciniphila* improves MASLD by regulating mitochondrial oxidation, bile acid metabolism, and L‐aspartate transport in the gut–liver axis [96]. The gut–liver axis integrates barrier function, microbial ecology, and metabolite signaling, becoming a key target for interrupting inflammation, metabolic disorder, and hepatic fat accumulation.

### 5.2. Gut–Brain Axis and Metabolic Comorbidities

Metabolic diseases (obesity, T2DM, and MASLD) are often combined with neuropsychiatric symptoms (cognitive decline, depression, and anxiety), involving the gut–brain axis [80]. Gut dysbiosis affects brain function through neural (vagus nerve), endocrine (HPA axis), immune (neuroinflammation), and metabolic (microbial metabolites) pathways [80]. High‐fat diet‐induced dysbiosis damages the intestinal barrier, increasing circulating inflammatory cytokines and LPS, which cross the blood–brain barrier or activate the vagus nerve, triggering neuroinflammation and neuroendocrine disorder, leading to cognitive and mood disorders [81]. Gut microbiota directly regulates neurotransmitter synthesis (GABA, serotonin, and dopamine), and tryptophan metabolism disorder affects mood and cognition [83]. TCM formulas targeting kidney‐yin deficiency improve diabetic cognitive dysfunction via the microbiota–gut–brain axis: the LLKL formula regulates gut microbiota, maintains intestinal epithelial homeostasis, and reduces LPS and proinflammatory cytokines (TNF‐*α* and IL‐6), protecting brain function [84]. The gut–brain–liver axis is a unified metabolic regulatory system: hepatic factors and microbial metabolites affect CNS function, and obesity/insulin resistance aggravates neuroinflammation and neurodegeneration [81]. The vagus nerve transmits signals of gut peptides (CCK, GLP‐1, PYY, and 5‐HT) to the brainstem, regulating appetite, mood, and metabolism [83]. TCM herb pairs (such as Ginseng Radix–Acori Tatarinowii Rhizoma) restore gut microbiota, strengthen the intestinal barrier, and regulate vagus nerve signaling and neurotransmitter synthesis, alleviating neuropsychiatric comorbidities of metabolic diseases, in line with the holistic concept of TCM.

### 5.3. Gut–Bone Axis and Metabolic Bone Disease

Emerging studies confirm that gut microbiota homeostasis is closely related to bone health, forming the gut–bone axis, which is involved in the pathogenesis of metabolic bone disease (osteoporosis). The main mechanisms are as follows: (1) SCFAs (acetate, propionate, and butyrate) produced by gut microbiota promote Treg differentiation, inhibit systemic inflammation, and reduce osteoclastogenesis induced by proinflammatory cytokines (TNF‐*α*, IL‐1*β*, and IL‐17) [85]; and (2) butyrate directly promotes osteoblast differentiation and inhibits osteoclast formation in vitro. TCM herb pairs for “tonifying kidney and strengthening bone” (such as Drynariae Rhizoma–Dipsaci Radix and Epimedii Folium–Morindae Officinalis Radix) may exert bone‐protective effects by regulating the gut–bone axis, acting as prebiotics to enrich SCFA‐producing bacteria (*Lactobacillus*, *Bifidobacterium*, and *Akkermansia*), increasing SCFA levels, activating GPR41/GPR43 on immune and bone cells, inhibiting osteoclast formation, and repairing the intestinal barrier to prevent bacterial component translocation and bone loss [82]. Bile acids and polyamines also regulate bone metabolism, but the mechanism needs further study. The therapeutic potential of TCM herb pairs for metabolic bone disease lies in correcting gut dysbiosis, increasing beneficial metabolites, restoring intestinal barrier function, and rebalancing immunity, favoring bone formation over resorption and realizing the holistic regulation of bone metabolism and systemic health.

## 6. Material Basis and Compatibility Rules of TCM Herb Pairs Regulating Gut Microbiota

### 6.1. Contribution of Characteristic Active Ingredient Groups

TCM herb pairs with different properties, flavors, and meridian tropisms contain specific active ingredient groups, which act on the gut microbiota in a targeted manner: (1) bitter–cold herb pairs (*C. chinensis*–*S. baicalensis*) are rich in alkaloids and flavonoids, with strong antibacterial and anti‐inflammatory effects, quickly correcting damp‐heat‐type gut dysbiosis [86]; and (2) sweet–warm herb pairs (*A. membranaceus*–*C. pilosula*) are rich in polysaccharides, promoting beneficial bacteria growth and enhancing intestinal barrier function [30]. These phytochemical groups form a complex interaction network in the intestinal lumen, producing synergistic effects. *Astragalus* polysaccharides selectively promote butyrate production through the synergistic effect of polysaccharide‐degrading bacteria and butyrate‐producing bacteria, which cannot be fully replicated by other polysaccharides [30]; flavonoids from *Amomum tsaoko* improve constipation by regulating gut microbiota and the serotonergic synapse pathway [88]. The multicomponent characteristic is crucial: the Yinchen Wuling powder (YCWLP) exerts antihepatic fibrosis effects through multiple active components metabolized by gut microbiota [89]. The regulation is bidirectional: herb pairs affect microbiota composition, and microbiota determines the biotransformation and bioavailability of herbal components. Antibiotics disrupting gut microbiota significantly reduce the plasma concentration of active ingredients (caffeic acid and formononetin) in Danggui Buxue Tang, reducing its efficacy [90]. *Polygonatum kingianum* polysaccharides improve lipid metabolism by regulating gut microbiota and miR‐484 [91]. The characteristic active ingredients of herb pairs interact dynamically with the gut ecosystem, reshaping microbial diversity and function and forming the material basis for improving metabolic diseases.

### 6.2. Synergistic Mechanism Based on “Jun–Chen–Zuo–Shi” Compatibility

In TCM herb pairs, the “Monarch” herb targets the main disease and syndrome, containing core active ingredients to regulate key gut microbiota or metabolic pathways; the “Minister” herb assists the Monarch, expanding the therapeutic scope or enhancing efficacy by regulating different bacteria or metabolic pathways, forming a multitarget synergistic network. For example, in the treatment of diabetes, *C. chinensis* (Huanglian) is the Monarch herb, clearing heat and drying dampness, inhibiting harmful bacteria, and its active component berberine inhibits the TLR4/NF‐*κ*B pathway [92]; when paired with *Cinnamomum cassia* (Rougui) (Minister herb), which warms and promotes circulation and moderates the cold nature of *C. chinensis*, its component cinnamaldehyde promotes SCFA‐producing bacteria growth, improving glucose metabolism from another dimension [93]. This synergy is reflected in the complementary regulation of microbial metabolites: SCFAs regulate immunity, inflammation, and glucose homeostasis [97]; the Monarch herb inhibits pathogens and inflammation, and the Minister herb promotes beneficial bacteria and SCFA production. Complex formulas such as the ZiBuPiYin recipe (ZBPYR) prevent diabetic cognitive decline by regulating gut microbiota and metabolites (SCFAs and branched‐chain amino acids), affecting hippocampal metabolism [98]; the modified Bufei decoction (MBFD) improves calf immunity and reduces pneumonia by regulating gut microbiota (increasing Bacteroidetes) [99]. Network pharmacology shows that the Hongjinshen decoction treats pulmonary fibrosis through multicomponent and multitarget regulation [100]. SLBZS synergizes with PD‐1 antibody immunotherapy for non–small cell lung cancer by remodeling gut microbiota (*Akkermansia* and *Lactobacillus*) and regulating SCFAs, enhancing antitumor immunity and reducing side effects [34]. The Minister herb can also reduce the side effects of the Monarch herb and improve bioavailability. Based on the microbiota–gut–organ axis, herb pairs improve MASLD by restoring microbial diversity, strengthening intestinal barrier function, and normalizing bile acid and SCFA metabolism [33]. The “Jun–Chen” compatibility logic provides a theoretical basis for designing herb pairs that interact with the gut microbiota and its metabolites, achieving better therapeutic effects than single herbs.

## 7. Application of Modern Research Techniques and Methods

### 7.1. Multiomics Integration Analysis Strategy

Multiomics integration (metagenomics, fecal/serum metabolomics, and transcriptomics) provides a systematic framework for revealing the mechanism of herb pairs in treating metabolic diseases, capturing the interaction between gut microbial gene function, host–microbiota co‐metabolites, and host tissue response [101]. Metagenomic sequencing reveals the functional changes of the gut microbiome (such as BSH and SCFA synthesis genes) [101]; metabolomics quantifies microbial metabolites (bile acids and SCFAs) [102]; transcriptomics detects host gene expression (inflammation, insulin signaling, and lipid metabolism) [103]. Advanced bioinformatics tools (correlation network analysis, machine learning, and AI) identify multiomics biomarkers [104]; iModMix discovers multiomics modules [105]; MultiBaC eliminates batch effects [106]. This strategy has been successfully applied in cancer research [107], and its workflow in herb pair research is as follows: (1) metagenomics identifies herb‐induced beneficial bacteria and functional pathways (SCFA production); (2) metabolomics confirms elevated SCFA levels; (3) transcriptomics verifies the upregulation of host genes (GLP‐1 secretion and PPAR*γ* activation); and (4) network pharmacology integrates multiomics data to map metabolic disease pathways [108]. This multilayer evidence chain confirms that the therapeutic effect of herb pairs is mediated by gut microbiota remodeling and metabolite regulation.

### 7.2. Germ‐Free (GF) Animals and Microbiota Transplantation Validation

GF animal models, antibiotic‐treated animals, and fecal microbiota transplantation (FMT) are the “gold standard” for verifying the mediating role of gut microbiota in herb pair efficacy [109]. GF animals have no gut microbiota; if herb pairs fail to improve metabolic phenotypes (glucose tolerance and hepatic steatosis) in GF animals, it proves that gut microbiota is necessary for their efficacy [109]. Antibiotic depletion of gut microbiota also abolishes the therapeutic effect of herb pairs. Conversely, FMT from herb‐treated donor animals to diseased recipient animals transfers the therapeutic effect (weight loss, improved insulin sensitivity, and reduced inflammation), proving that the altered microbiota itself is the key factor [110]. This strategy separates the effect of microbiota from the direct pharmacological effect of herbal components. For example, herb pairs enrich *A. muciniphila* and butyrate‐producing bacteria, and FMT transfers these bacteria to recipients, improving metabolic parameters, which can be verified by multiomics [111]. Advanced applications include humanized gnotobiotic mice (colonized with human microbiota) [112], and FMT combined with multiomics identifies key microbial strains and metabolites [113]. This causal verification framework is crucial for translating TCM herb pairs into modern mechanism‐based therapies, moving beyond correlation to causality.

## 8. Current Challenges and Future Prospects

### 8.1. Research Depth and Standardization Dilemma

Current studies on TCM herb pairs regulating gut microbiota to improve metabolic diseases mostly focus on descriptive correlations, lacking clear causal mechanisms [29], [83], [114]. The chemical complexity of herb pairs leads to unclear interaction networks between phytochemicals, microbial biotransformation, and host response [26–113, 115–130], and the specific pathways, key bacteria, and host axis (gut–liver and gut–brain) mechanisms are not clearly elucidated [23], [33], [131]. In addition, the lack of standardization in botanical sources, processing (paozhi), extraction, and dosage leads to poor reproducibility of research results [33]. Preclinical studies over‐rely on male C57BL/6 mice (68% in MASLD studies) [33], which cannot simulate human genetic, dietary, and microbial diversity. Most studies use 16S rRNA sequencing, which only provides taxonomic information but lacks functional metabolic data (bile acids and SCFAs) [29–31, 33–113], [117–130, 132, 133]. Therefore, future research needs to shift from correlation to causality and adopt chemically standardized herb pairs, advanced multiomics, and more clinically relevant disease models.

### 8.2. Clinical Translation and Personalized Therapy

The clinical translation of TCM herb pairs targeting gut microbiota is limited by the lack of high‐quality human studies: there are few large‐scale randomized controlled trials (RCTs) with histological endpoints (liver biopsy) or hard clinical outcomes, and only 4% of MASLD herb pair studies progress to clinical trials [33]. Clinical studies rely on 16S rRNA sequencing, lacking functional microbiota characterization [29–31, 33–69, 72–79], [93], [117–119], which is crucial for linking microbial changes to host metabolism [27–69, 72–79], [93], [115–127]. Future personalized therapy strategies are as follows: (1) establish a “syndrome (Zheng)–microbiota” typing system, combining TCM syndrome differentiation (spleen deficiency and damp‐heat) with gut microbial and metabolic signatures, guiding personalized herb pair selection [114]; and (2) apply synthetic biology to develop engineered probiotics, which can biotransform TCM prodrugs into active metabolites or secrete therapeutic molecules (SCFAs and anti‐inflammatory cytokines) in the gut, synergizing with herb pairs to improve bioavailability and reduce side effects [33]. Integrating syndrome–microbiota diagnosis and engineered microbial therapy will transform TCM herb pairs from broad‐spectrum mixtures into precision‐targeted therapies, bridging traditional wisdom and modern precision medicine.

## 9. Conclusion

The interaction between TCM herb pairs and gut microbiota is a new paradigm for understanding their therapeutic effects on metabolic diseases. The multicomponent synergy of herb pairs forms a bidirectional “microbial dialogue” with the gut ecosystem, which is the core mechanism of their holistic multitarget regulation. This mechanism includes the following: reshaping gut microbial community structure; regulating key microbial metabolites (SCFAs, bile acids, and tryptophan metabolites); repairing intestinal barrier function and alleviating metabolic endotoxemia; and regulating the microbiota–gut–organ axis (gut–liver, gut–brain, and gut–bone) crosstalk. This integrates the ancient “Jun–Chen–Zuo–Shi” compatibility theory with modern microbiome science, explaining the systemic metabolic regulation of orally administered herb pairs.

At present, the field has confirmed the correlation between herb pairs, gut microbiota, and metabolic improvement but faces challenges such as unclear causal mechanisms, lack of standardization, and insufficient clinical translation. Future research needs to use multiomics, GF animals, FMT, and other technologies to clarify the causal chain: herb pair → signature active components → core functional microbiota → key metabolites → host targets (FXR, TGR5, and GPRs) → phenotypic improvement. At the clinical level, large‐scale RCTs, chemical fingerprinting, and baseline microbiome characterization are needed to develop personalized “syndrome–microbiota” precision therapy.

In summary, the research on TCM herb pairs and gut microbiota is a promising interdisciplinary field, which can not only reveal the scientific connotation of TCM but also develop new microbiota‐targeted strategies for the prevention and treatment of global metabolic diseases.

## Funding

This work was supported by the 2026 “An Xin Qi Yan” Project of Dan’an Academy, Nanjing University of Chinese Medicine, and the College Students’ Innovation Training Program of Jiangsu Province (Project No. X2026103150135: Exploration of Syndrome Differentiation Rules of Herbal Pairs for Thyroid Diseases by National TCM Master Zhou Zhongying and Development of an Agent‐Assisted Decision‐Making System).

## Conflicts of Interest

The author declares no conflicts of interest.

## Data Availability

The data that support the findings of this study are available on request from the corresponding author. The data are not publicly available due to privacy or ethical restrictions.
